# Genome Sequences of Coxsackievirus B5 Isolates from Two Children with Meningitis in Australia

**DOI:** 10.1128/genomeA.01125-17

**Published:** 2017-10-12

**Authors:** Bixing Huang, Bruce Harrower, Peter Burtonclay, Tanya Constantino, David Warrilow

**Affiliations:** Public Health Virology Laboratory, Queensland Health Forensic and Scientific Services, Coopers Plains, Queensland, Australia

## Abstract

Two coxsackievirus B5 (CVB5) strains were isolated from two children with aseptic meningitis in Australia. Their genomes were sequenced and found to be divergent from the previously reported CVB5 genome sequences, with both having 84% and 97% identities to the closest strains at the nucleotide and amino acid levels, respectively.

## GENOME ANNOUNCEMENT

Coxsackievirus B5 (CVB5) is a serotype strain of the species *Enterovirus B* of the genus *Enterovirus* in the family *Picornaviridae* (1). It is a nonenveloped virus with a single-stranded positive RNA genome of approximately 7.4 kb in length. The genome has a long 5′ untranslated region (UTR) containing a type I internal ribosome entry site (IRES) and a polyadenylated tail at the 3′ terminus. The genome has a single open reading frame (ORF) encoding a polyprotein. The 5′ P1 region encodes the structural proteins, and the P2 and P3 regions encode nonstructural proteins involved in viral replication (2). CVB5 is associated with cases of aseptic meningitis, encephalitis, myocarditis, and some chronic diseases (2). It is one of the most commonly reported serotypes of enterovirus infection in Australia (3). Similar to other enteroviruses, CVB5 is genetically highly diverse (2). Here, we report the sequences of two novel CVB5 strains from Queensland, Australia, which were divergent from the CVB5 genomes reported previously.

Cases 1 and 2 were children aged 21 months (female) and 1.5 months (male), respectively, with neurological manifestations. No epidemiological links were evident, as their residences were separated by more than 1,000 km, and they were admitted to different hospitals more than 1 month apart. Cerebrospinal fluid (CSF) specimens taken from each patient were bacterial culture negative but enterovirus RNA positive by reverse transcription-PCR (RT-PCR). Buffalo green monkey kidney (BGMK) cells were used for virus culture, and an isolate was obtained from each CSF specimen (4). The supernatants were extracted for viral nucleic acids with a QIAamp viral RNA extraction kit (Qiagen) without carrier RNA. Illumina sequencing was performed as previously described (5). Genome consensus sequences were assembled using the Geneious R8.1 software at the lowest sensitivity settings (6).

The sequence of the first strain, designated AU17EV1, was assembled using a *de novo* approach, and the contigs were mapped to a coxsackievirus B1 reference genome (NCBI reference sequence NC_001472) (7). The online Enterovirus Genotyping Tool (http://www.rivm.nl/mpf/typingtool/enterovirus/) was used for genotyping, where it was assigned CVB5 (8). The strain isolated from case 2 was designated AU17EV2. Its genome was assembled using the AU17EV1 sequence as a reference, and it was assigned to CVB5 as well. The complete coding drafts were both 7,378 nucleotides (nt), excluding the 3′ poly(A) tail, and were 99.1% identical at the nucleotide level. Their GC contents were 47.2% (AU17EV1) and 47.4% (AU17EV2). The genomes encoded a single 2,185-amino-acid polyprotein. Their polyproteins shared 99.5% amino acid identity.

The predicted polyprotein of AU17EV1 had the best match to CVB5 strain 84-6500/France/84 (GenBank accession number KT285016), with 97% identity (9). The predicted polyprotein of AU17EV2 had the best match to CVB5 strain 4634/83/USA (accession number KT285009), with 97% identity (9). Both genome sequences shared the highest nucleotide identity (84%) with CVB5 strain 1954/UK/85 (accession number X67706), with 84% identity (10). The result suggested that the two CVB5 genomes were significantly divergent from previous nucleotide genomic sequences available in GenBank.

### Accession number(s).

The two near-complete genome sequences were deposited into GenBank under accession numbers MF683838 (AU17EV1 strain) and MF683839 (AU17EV2 strain).
